# “Parasitic Weeds: Biology and Control” Special Issue Editors Summary

**DOI:** 10.3390/plants11141891

**Published:** 2022-07-21

**Authors:** Evgenia Dor, Yaakov Goldwasser

**Affiliations:** 1 Institute of Plant Protection, Newe Ya’ar Research Center, Agricultural Research Organization, P.O. Box 1021, Ramat Yishay 30095, Israel; 2 The Robert H. Smith Institute of Plant Sciences and Genetics in Agriculture, Faculty of Agriculture, Food and Environment, The Hebrew University of Jerusalem, P.O. Box 12, Rehovot 76100, Israel

We are happy to summarize this important Special Issue (SI) of MDPI *Plants*—“Parasitic Weeds: Biology and Control”.

Parasitic plants are scientifically interesting and agriculturally important weeds which are spreading worldwide with limited control means. Plant parasitism is a case of extreme plant-to-plant interactions when parasitic plants connect directly to the vasculature system of a host plant, extracting water and nutrients from them, and assimilates [1]. During the evolution from non-parasitic origin, parasitic plants have developed many specific functions, such as host detection, host attachment, host exploitation, and host defense suppression. The world of parasitic plants includes about 20 families, with a wide trophic spectrum from facultative hemiparasites, which are able also to perform photosynthesis and therefore may survive without a connection to the host, to obligatory holoparasites, which have no photosynthetic abilities [2]. Some parasitic species are noxious weeds and damage major agricultural crops, causing heavy economical losses worldwide [3,4].

The parasitic lifestyle in plants has always been the subject of curiosity of scientists, but during the last decade our understanding of parasitic plant—host interaction has greatly evolved due to rapid advances in molecular and genomic tools, especially such as high throughput DNA sequencing, transcriptomics and metabolomics. The latest findings take the science of parasitic plants to a higher level and open up new horizons in parasitic plant management. The discovery of a novel family of phytohormones, the strigolactones [5,6], and their involvement in host detection and evolution of parasitic plants [7,8], the detection of the information exchange between host and parasite [9]; and the elucidation of the host defense mechanisms suppression by the parasites [10] have led to a deeper understanding of physiological processes in host-parasite interaction. In the light of recent achievements, the re-evaluation of control management, including crop breeding, and molecular genetics is on the agenda. 

Finally, 11 papers were collected for the SI; of them, five original research papers present new strategies in parasitic weeds management [11,12,13,14,15], two focused on crop resistance to parasitic plants [16,17] and three provide new insights on plant—parasite interaction [18,19,20]. One Opinion Paper provides a personal view of the present status of parasitic weed problems and their control written by Chris Parker, who dedicated his entire long scientific career to parasitic plants and their management [21].

We thank all the authors for their valuable articles. We are especially proud of the active participation of young scientists in our Special Issue. Four articles were submitted for participation in the student paper competition [11,14,15,17]. The winner of the competition was the Ph.D. student Dana Sisou from the Department of Phytopathology and Weed Research, ARO, Newe Ya’ar Research Center. The title of her paper was based on her Ph.D. thesis: Biological and transcriptomic characterization of pre-haustorial resistance to sunflower broomrape (*Orobanche cumana* W.) in sunflowers (*Helianthus annuus*) [17].

We would particularly like to acknowledge Chris Parker’s paper contribution, the parasitic plant elder on one end, and the inclusion of student papers on the other end—we feel that we have successfully encouraged both and those in between to contribute to this important field. 

Finally, we offer special thanks to Mrs. Sumi Sun, the MDPI *Plants* Special Issue coordinator for her patience, good work, and assistance with the editing and publication processes. 
